# Measurement of Pancreatic Stone Protein Compared with C-Reactive Protein and Procalcitonin in the Diagnosis of Sepsis in an Intensive Care Unit: A Systematic Review

**DOI:** 10.21315/mjms2024.31.5.3

**Published:** 2024-10-08

**Authors:** Septian Adi Permana, Purwoko, Enrico Jonathan Hartono

**Affiliations:** Department of Anesthesiology and Intensive Therapy, Dr. Moewardi General Hospital, Surakarta, Indonesia

**Keywords:** C-reactive protein, diagnosis, intensive care, pancreatic stone protein, procalcitonin, sepsis

## Abstract

Sepsis remains a significant challenge in the intensive care unit (ICU), with prompt diagnosis and management being critical to improving patient outcomes. Biomarkers have emerged as valuable tools for identifying and predicting sepsis outcomes, with pancreatic stone protein (PSP), procalcitonin (PCT) and C-reactive protein (CRP) as three promising candidates. This systematic review aimed to analyse and compare the diagnostic accuracy of PSP, PCT and CRP regarding sepsis in the ICU. A review of the literature on the diagnostic performance of the three biomarkers was performed using PubMed Central, PubMed, ScienceDirect, Oxford Academic, SpringerLink and Cochrane Library. Data regarding the diagnostic accuracy of the three biomarkers were extracted, compared, and represented as the area under the curve (AUC) receiver operating characteristics (ROC). Three studies examining PSP, PCT and CRP biomarkers in 858 adult patients admitted to the ICU were included in this review. Compared with PCT and CRP, the PSP biomarker, with its unique applications and properties that may potentially benefit patients, doctors and hospitals, performed well and proved reliable in diagnosing sepsis in adult patients. PSP demonstrated reliability in sepsis diagnosis. Further analysis should be conducted to establish a formal, appropriate indication, as well as to determine a suspected sepsis patient’s condition when testing each biomarker.

## Introduction

Sepsis is a serious, life-threatening medical condition characterised by a host’s uncontrolled immune response to infection, which leads to dysfunction in multiple organs (1). Sepsis and septic shock remain global health problems associated with high morbidity and mortality (2), affecting more than 30 million people per year globally and are the leading causes of mortality in the pediatric population worldwide, accounting for 5 million deaths per year (1, 3). Sepsis is the primary cause of hospital readmissions, lasting impairments and reduced quality of life, which leads to a higher risk of mortality in the long term (1, 4). When dealing with acute infections and suspected sepsis patients in emergency departments, hospitals usually rely on general practitioners (GPs). GPs are in charge of first-hand decision-making regarding a patient’s needs regarding immediate hospital care and discharge (5, 6). This initial step of recognising and managing sepsis early on significantly influences the later outcomes of patients with sepsis (1).

There are three biomarkers that aid in diagnosing sepsis: i) C-reactive protein (CRP), ii) pancreatic stone protein (PSP) and iii) procalcitonin (PCT) (5). CRP is a well-known marker of inflammation and is widely used to assist in diagnosing infections, while PCT is relatively new (5, 6) and has undergone extensive evaluation over the last two decades as a marker of bacterial infection (5, 6). Despite their common use in sepsis diagnosis, both CRP and PCT have their own shortcomings (6, 7).

The PSP biomarker is a type of lectin protein that activates polymorphonuclear cells and exhibits proinflammatory activity in laboratory settings (8). It is a novel biomarker for infections that has been thoroughly evaluated in various patient groups and clinical settings, including emergency rooms and intensive care units (9). In a study of critically ill adults, the PSP biomarker outperformed PCT and other sepsis biomarkers to accurately identifying sepsis (2) and it can also be used as a predictor of mortality in the ICU (10). Additionally, point-of-care testing of CRP and PCT are not routinely done in the ICU, while PSP can be measured routinely and at bed-site within 5 min using a single drop of blood, allowing a more simple and frequent biomarker assessments (11). The PSP biomarker is not only being used for diagnosis but also to asses severity and predicts patient outcome (2). Nevertheless, the establishment of a clinically significant threshold level for PSP remains unresolved (5).

Our objective was to conduct an individual patient-level systematic review of existing data to assess the performance of PSP compared to PCT and CRP for sepsis diagnosis in the ICU.

## Method

### Search Strategy and Selection Criteria

A comprehensive systematic search was conducted per PRISMA (Preferred Reporting Items for Systematic Reviews and Meta-Analyses) guidelines. Scientific databases including PubMed Central, PubMed, ScienceDirect, Oxford Academic, SpringerLink and Cochrane Library were searched. The keywords used to search from the journals are ‘sepsis’, ‘procalcitonin, ‘pancreatic stone protein’, ‘PSP’, ’c-reactive protein’, ‘CRP’, ‘intensive care’ and ‘ICU’. The inclusion criteria for this study encompassed cohort studies that employed PSP, PCT and CRP to establish sepsis diagnoses in adult patients who had not previously received a sepsis diagnosis in the ICU. Reviewers excluded pediatric cohort/trials, study protocols and guidelines. Each reviewer manually extracted the area under the curve (AUC) receiver operating characteristics (ROC) data, which represents the accuracy of sepsis diagnosis, from the relevant studies for further comparison in our research.

It became evident that the resulting pool of eligible studies was insufficient in terms of quantity to warrant a comprehensive meta-analysis. Given the limited number of studies meeting our stringent criteria, it is prudent to acknowledge that conducting a meta-analysis would be impractical and potentially yield inconclusive results. Therefore, this study is proceeded in a systematic review without meta-analysis or the implementation of SWiM (Synthesis Without Meta-analysis) manner using a guideline provided by SWiM Project Team (swim.sphsu.gla.ac.uk). This systematic review protocol has been submitted to PROSPERO (CRD42023421501)

### Outcome

The primary outcome was the sepsis diagnosis assessed by PSP, PCT and CRP levels.

### Quality Assessment

The Newcastle-Ottawa Quality Assessment Scale for Cohort Studies was used for evaluating included studies. Each reviewer independently evaluates the three domains of quality assessment process: i) selection, ii) comparability and iii) outcome.

## Results

### Study Selection

**Figure 1:**
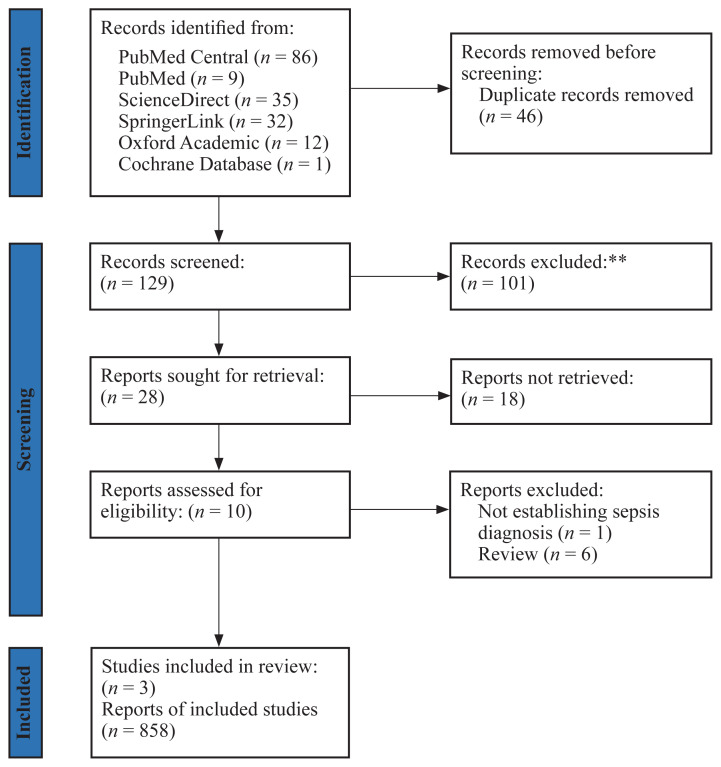
Study selection

A total of three studies were identified after excluding duplicates, pediatric studies, review and guideline studies/study protocol (Figure 1). The final 3 studies accounted for 858 participants in total. A total of 18 studies were not included in this review due to inability to retrieve the full text manuscripts.

In this study, the three specific biomarkers (PSP, PCT and CRP) are being compared regarding the different performance of each biomarker to establish sepsis diagnosis in ICU. The complete study information is addressed and can be viewed in Table 1.

The Newcastle-Ottawa Quality Assessment Scale for Cohort Studies were used to assess the quality of each included literatures examined in current study. Among all cohort studies, Pugin et al. (10) scored the best and Parlato et al. (14) scored the lowest. All studies examined in Table 2 are qualified as good with no bias in data selection, good comparibility of cohort groups and good assessment of the outcome.

### Characteristics of Included Studies and Participants

The characteristics of the three studies in this review are summarised in Table 3. The clinical sepsis diagnosis establishment process was different among the three studies (due to the patient’s variable presenting symptoms) yet the biomarker tests within all the studies were similarly examined. The patient population are categorised as presenting with infection or without infection prior to sepsis.

The three biomarkers were not only being used to establish a new sepsis diagnosis but also to predict a septic event and evaluation. While not every literature had similar charateristics among the subjects, all of the study comparably evaluates diagnostic accuracy of each biomarker with the same parameters, the AUC ROC values.

## Discussion

Prompt sepsis diagnosis is important due to its precarious disease progression. In the ICU setting, sepsis alone contributes to 30% of mortality globally and increases with complications to 50% of cases (3). Hence, the question of choosing the most useful tools to diagnose sepsis is key. Still, numerous options of tools to diagnose sepsis are available with each have a distinct use case (6).

A ROC curve plays a central role in this diagnostic process. It serves as an analytical tool presented graphically, employed for assessing the performance of binary diagnostic classification methods (12). To apply this method, diagnostic test outcomes, often expressed as continuous or ordinal variables, must be categorised into distinct binary categories, typically indicating the presence or absence of a disease (12). The AUC, widely utilised to assess the accuracy of diagnostic tests, offers an effective combined measure of sensitivity and specificity, conveying the inherent validity of these tests (12, 13). The ROC curve links data points by utilising specificity (false positive rate) on the x-axis and sensitivity (true positive rate) on the y-axis, encompassing all cutoff values derived from the test outcomes (12). When the standards for classifying a positive result become more stringent, the curve exhibits a trend of shifting downwards and towards the left (more specific), reflecting this increased stringency in the diagnostic criteria. Conversely, when a lenient standard is employed, the point on the curve shifts upwards and towards the right (more sensitive) (12).

For a meaningful diagnostic technique, AUC should exceed 0.5 and typically surpass 0.7 for fair acceptability (12, 13). When comparing multiple diagnostic tests, the ROC curve with the highest AUC is deemed superior in diagnostic performance (12). It is often accompanied by a 95% confidence interval (CI) due to the influence of statistical errors on the data, providing a range of potential values around the actual AUC value (12).

The focus in this review was on comparing biomarkers as a modality for diagnosing sepsis. It is found that generally the three reviewed biomarkers have a positive correlation between sepsis diagnosis and positive test results observed by the value of AUC ROC obtained for each of the included studies. This proves the usefulness of the three biomarkers in the interest of establishing sepsis diagnosis.

The use of CRP as a biomarker to help diagnose and treat sepsis has been documented in many studies (14). Regular use of CRP is found to be successful in improving antibiotic therapy in critically ill patients by decreasing treatment duration (15). However, based on prior studies, CRP does not have a consistent level of accuracy in sepsis diagnosis (14, 16). It may have been because of CRP’s nature as an acute response protein, hence when exposed to a diverse unique situation of testing it was found hard for the biomarker to endure (16). An alternative, more stable to an actual septic, has been in dire need to be proposed.

Studies had already shown the specificity and sensitivity among the most used biomarkers in patients with suspected or confirmed sepsis diagnosis. In one study, the differences in diagnostic value for a total of eight biomarkers (CRP, lactate, PCT, high sensitivity troponin I, N-terminal pro-b-type natriuretic peptide, creatinine, urea and PSP) were analysed (17). Loots et al. (17) conducted the same study by comparing the sensitivity and specificity between biomarkers using ROC curve and calculating the C statistic (area under the ROC curve) after obtaining the sensitivity and specificity values for different cut-offs points. Based on supplementary Table 1 attached by Loots et al.’s (17) study, in respect of PCT, CRP and PSP cut-offs, the most balanced number between the sensitivity and specificity was procalcitonin > 0.25 ng/mL (sensitivity of 51% and specificity of 79%), CRP > 100 mg/L (sensitivity of 40% and specificity of 72%) and pancreatic stone protein < 100 ng/mL (sensitivity of 71% and specificity of 37%). From the ROC curves, it was also shown that procalcitonin line graphs were positioned above the reference curve and pancreatic stone protein’s curve with CRP’s curve in the middle in the same study (17). The position of the graph is influenced by the number of false positive test results in several pre-determined cutoffs, where according to the order of the graph, the lowest false positive rate is for the procalcitonin biomarker, followed by CRP and lastly PSP (17).

Although PSP was considered to have a poorer performance compared to PCT to establish a sepsis diagnosis, another study exhibited a novel use of measuring PSP when used sequentially (10). The higher sensitivity rates of PSP were taken advantage of to predict a sepsis event (10).

Pugin et al. (10) conducted a cohort study design with unselected critically ill patients without an initial history of sepsis diagnosis in the ICU. They observed the patient as the disease progressed and investigated the clinical and diagnostic test results (including biomarkers) until the sepsis diagnosis was established. However, it has to be addressed that sepsis diagnosis was not the same as the sepsis event (10). The prior researchers suspected that sepsis events could occur before a sepsis diagnosis can be established, therefore, the researchers also formed an independent committee (composed of three ICU experts) to retrospectively review the case, then furthermore it was validated whether the patient had experienced a septic event while staying in the ICU prior to sepsis diagnosis (10).

In respect of PCT, CRP and PSP median time interval (between the septic event) and clinical diagnosis of sepsis was established, pancreatic stone protein can ‘predict’ sepsis 5 days (*P* = 0.003) prior while procalcitonin can ‘predict’ sepsis 3 days (*P* = 0.025) prior the sepsis diagnosis was established (10). CRP levels, however, were beginning to raise 2 days prior sepsis diagnosis (*P* = 0.009). This prediction was made in regards to when (how many days before sepsis diagnosis) the levels started to increase (10).

This present study has several limitations that should be acknowledged to provide a comprehensive understanding of its findings. Firstly, the study solely focuses on evaluating diagnostic accuracy using the AUC ROC. While AUC ROC is a valuable metric for assessing the performance of diagnostic tests, it should be noted that it represents a single perspective in the evaluation process. Further study still needed, preferably that utilise other metric such as positive or negative predictive value to diagnose sepsis.

## Conclusion

Sepsis remains a major challenge in the ICU, requiring prompt diagnosis and appropriate management to improve patient outcomes. CRP, PCT and PSP are three biomarkers that have shown promise in the diagnosis and prognostication of sepsis. While the three biomarkers have demonstrated high sensitivity and specificity in several studies, their clinical utility may depend on various factors, such as patient population, disease severity and comorbidities. CRP, PCT and PSP offer benefits that are unique in certain aspects and may be useful not only to diagnose but to improve patient care among individuals with or suspected sepsis. However, future research should focus on optimising the use of these biomarkers to improve the accuracy of sepsis diagnosis and risk stratification in the ICU, ultimately leading to better patient outcomes.

## Figures and Tables

**Table 1:** Study characteristics

Reference	Study design	Period of data collection	Country	Clinical condition	Participant number (*n*)	Patients’ age, median	Male gender %	Female gender %	ICU LOS (days)*
Pugin et al. (10)	Multicentre, prospective blind cohort	June 2018–March 2019	France, Switzerland, Italy and United Kingdom	Unselected ICU patients	243	65	63	37	9
Loots et al. (17)	Prospective blind cohort	June 2018–March 2020	Netherlands	Sepsis within 72 h in ICU	336	79	60	40	4.7
Parlato et al. (14)	Multicentre, prospective blind cohort	December 2011–April 2013	Paris, France	ICU patients with sepsis or non-septic SIRS	279	65	63	37	–

Note:

* median;

ICU LOS = Intensive care unit length of stay (in days); SIRS = Systemic Inflammatory Response Syndrome

**Table 2:** Quality assessment for literatures using Newcastle-Ottawa Quality Assessment Scale for Cohort Studies

Reference	Selection^a^				Comparibility^b^	Outcome^c^			Total number of stars
	
	Representativeness of the exposed cohort	Selection of the non-exposed cohort	Ascertainment of exposure	Incident disease		Assessment of the outcome	Length of follow-up	Adequacy of follow-up	
Pugin et al. (10)	A*	A*	A*	A*	A**	A*	A*	B*	9
Loots et al. (17)	A*	A*	A*	B	A**	A*	A*	A*	8
Parlato et al. (14)	A*	A*	A*	B	A**	A*	A*	D	7

Notes:

a Selection
Representativeness of the exposed cohort: A. truly representative; B. somewhat representative; C. selected group; D. no description of the derivation of the cohort Selection of the non-exposed cohort: A. drawn from the same community as the exposed cohort; B. drawn from a different source; C. no description of the derivation of the non-exposed cohort. Ascertainment of exposure: A. secure record; B. structured interview; C. written self-report; D. no description. Absence of outcome in the beginning of study: A. yes; B. no.

b Comparability: To ensure that the cohorts are comparable based on their design or analysis methods: A. study controls for co-morbidities; B. study controls for any additional factor (e.g. age and severity of illness); C. not done.

c Outcome
Outcome assessment: A. independent blind assessment; B. record linkage; C. self-report; D. no description. Was follow-up long enough for outcomes to occur? A. yes, (i.e., in-hospital or up to 30 days); B. no. Adequacy of follow-up of cohorts: A. complete follow-up and all subjects accounted for; B. subjects lost to follow-up was unlikely to introduce bias, C. follow-up rate 90% or lower with no description of those lost; D. no statement.

Stars are allocated to each scoring category, with a maximum of four stars for selection, two stars for comparability and three stars for outcome. A study is considered to be of good quality if it earns 3 or 4 stars in the selection category, 1 or 2 stars in the comparability category, and 2 or 3 stars in the outcome/exposure category. Fair quality is indicated by a study receiving 2 stars in the selection category, 1 or 2 stars in the comparability category, and 2 or 3 stars in the outcome/exposure category. A study is deemed to be of poor quality if it has 0 or 1 star in the selection category, 0 stars in the comparability category, or 0 or 1 star in the outcome/exposure category.

**Table 3:** Quality assessment for literatures using Newcastle-Ottawa Quality Assessment Scale for Cohort Studies

Reference	Study purpose	Infection (*n*)	Non-infection (*n*)	Population characteristics	Measurement	SOFA score*	AUC-ROC Data			Study summary

							PSP	PCT	CRP	
Pugin et al. (10)	Evaluation of serial PSP and PCT levels for early sepsis detection	53	190	ICU patients without prior infection and no sepsis diagnosis	Accuracy (AUC-ROC) for sepsis versus no sepsis group	6 (5, 9) *P* < 0.05	0.75 (95% CI: 0.67, 0.82)	0.75 (95% CI: 0.68, 0.82)	0.77 (95% CI: 0.69, 0.84)	Similar diagnostic accuracy across PSP, PCT, and CRP
Loots et al. (17)	Comparison of sepsis-related biomarkers to clinical diagnostic model for sepsis diagnosis	141	195	ICU patients critically ill and experiencing fever, confusion, decline in health, or severe infection		–	0.57 (95% CI: 0.49, 0.63)	0.71 (95% CI: 0.65, 0.76)	0.60 (95% CI: 0.54, 0.66)	No added diagnostic value in biomarkers compared to diagnostic model (based on clinical and patient’s symptoms)
Parlato et al. (14)	Evaluation of sepsis-related biomarkers to differentiate sepsis diagnosis with non-septic SIRS	188	91	ICU patients with hypo- and hyperthermia and at least another SIRS criterion considered for antimicrobial therapy		9 (8, 10) *P* > 0.05	0.63 (95% CI: 0.54, 0.71)	0.55 (95% CI: 0.47, 0.62)	0.73 (95% CI: 0.65, 0.81)	CRP performs the best among tested biomarkers

Note:

* Median (Q1, Q3);

SOFA = sequential organ failure assessment; SIRS = Systemic Inflammatory Response Syndrome; ICU = intensive care unit; CRP = C-reactive protein; PSP = pancreatic stone protein; PCT = procalcitonin; AUC-ROC = Area under curve-receiver operating curves; CI = confidence interval
